# Early dopaminergic replacement treatment initiation benefits motor symptoms in patients with Parkinson's disease

**DOI:** 10.3389/fnhum.2024.1325324

**Published:** 2024-05-09

**Authors:** Xin Li, Zhao-ying Dong, Meng Dong, Lei Chen

**Affiliations:** ^1^Tianjin Medical University, Tianjin, China; ^2^Department of Neurology, Tianjin People's Hospital Tianjin Union Medical Center, Tianjin, China; ^3^Department of Neurology, Tianjin Huanhu Hospital, Tianjin, China; ^4^Tianjin Key Laboratory of Cerebrovascular Diseases and Neurodegenerative Disease, Tianjin, China

**Keywords:** Parkinson's disease, motor symptoms, non-motor symptoms, dopaminergic replacement treatment, treatment initiation

## Abstract

**Background:**

Parkinson's disease (PD) generally progresses slowly, but it is controversial whether delaying treatment accelerates the progression.

**Objective:**

Determine the correlation between the time of dopaminergic replacement treatment initiation and the severity of clinical symptoms in PD, including motor and non-motor symptoms.

**Methods:**

PD patients were divided between 155 people who were diagnosed *de novo* and 165 PD patients receiving dopamine replacement therapy. Basic patient characteristics included gender, age, age at onset, disease duration, and the time of dopaminergic replacement treatment initiation. We used MDS-UPDRS scores to evaluate the severity of motor symptoms and we also used the scale to assess the severity of non-motor symptoms such as cognition, mood, sleep, and quality of life.

**Results:**

The mean time between symptom onset and the initiation of drug treatment was 31.0 (22.5) months. After adjusting for age, sex, age at onset, and disease duration, we found that the MDS-Unified Parkinson's Disease Rating Scale (UPDRS)-III score increased faster in the *de novo* group with a similar disease duration (F = 8.7, *p* = 0.0034) than the treatment group. The cumulative incidence of progression to H-Y score 3 in de novo PD group over disease duration was 39.7% in 50months and 92.2% in 100 months, while in treated group such cumulative incidence was 15.5% in 50 months, 51.4% in 100 months and 81.5% in 150 months. The cumulative incidence of patients in the *de novo* PD group was higher than that in the treated group (*p* = 0.001), suggesting that untreated patients were more likely to progress to the advanced stages. Symptoms onset, the time between symptom onset and treatment initiation, age, sex, and disease duration explained 28.95% of the total variation in the MDS-UPDRS-III score for motor symptoms. In drug-naïve patients, the time between symptom onset and treatment initiation explained 20.1% of the total variation in the MDS-UPDRS-III score for motor symptoms (*t* = 6.15, *p* < 0.001).

**Conclusions:**

These data in our study showed that early dopaminergic replacement treatment have played a positive role in PD patients, while dopaminergic replacement delayed treatment might be detrimental to motor symptoms and non-motor state of PD patient. Recognizing early stage symptoms of PD and early diagnosis are of great significance to treatment.

## Introduction

Parkinson's disease (PD) is the second most common neurodegenerative disease and is characterized by motor symptoms such as bradykinesia, rigidity, and resting tremor. However, PD disease typically progresses slowly (Palma, 2018). Patients with PD also suffer from a wide range of non-motor symptoms such as olfactory dysfunction, orthostatic hypotension, rapid eye movement sleep behavior disorder (RBD), pain, depression, and cognitive decline (Kalia and Lang, 2015). PD is pathologically characterized by the loss of dopaminergic neuron in the substantia nigra (SN) and the presence of α-synuclein protein accumulation. PD has a prevalence in people aged 65 and above of about 1.7% (Li et al., 2023). One study has predicted that by 2030, the number of PD patients will reach 4.94 million in China, accounting for about 50% of the global incidence (Yue et al., 2024).

The main treatment options for PD are dopamine replacement strategies, including the dopamine precursor levodopa and dopamine receptor agonists (DA), and in drug-refractory cases, deep brain stimulation is used for treating PD patients (Verschuur et al., 2019). There are many available options and treatments for PD in clinical practice, but there are conflicting ideas about whether dopamine replacement should be initiated as early as possible or whether it should be delayed until the disease progression requires it to be initiated. Studies of the risks and benefits of early or delayed treatment have reach conflicting conclusions, with some evidence indicating greater benefits of early treatment (Lohle et al., 2014) and some evidence showing no effect of early intervention on disease progression (Barbagallo and Quattrone, 2019). Asimakopoulos et al. (2008) found that self-reported health status [Parkinson's Disease Questionnaire (PDQ-39)] did not change between PD patients receiving treatment or not. In their study 42 patients with PD were followed-up for 2 years, of whom 26 started treatment during the first year and 16 remained untreated. Those receiving treatment had significantly higher UPDRS and PDQ-39 scores at baseline. There was no significant deterioration in PDQ-39 score in either group (median change untreated 0.8 vs. treated 4.0; *p* = 0.47), despite a significant difference in the change in motor UPDRS scores (untreated 6.0 vs. treated −6.0; *p* = 0.03).

Compared with other oral pharmacotherapies, levodopa and DA are the most effective drugs for motor symptoms of PD (de Bie et al., 2020). However, due to concerns about side effects such as motor fluctuations, dyskinesia, and impulsive control disorders, their application is often delayed in the early treatment of PD. In China, some patients do not receive systematic treatment for a long time after the onset of PD symptoms due to various reasons, including personal beliefs (e.g., fear of side effects or greater trust in traditional Chinese medicine), the cost of treatment, and delays in diagnosing the disease (Zhang et al., 2014).

Therefore, in this study, we aimed to investigate whether the early treatment of dopamine replacement strategies confers a positive effect on PD motor and non-motor symptoms and subsequently improves daily living quality for PD patients using a cross-sectional study.

## Materials and methods

### Participants

This study involved 320 PD patients (160 men and 160 women). All of them were diagnosed by a movement disorders specialist (Chen L). In this group, 155 patients were *de novo* PD patients who were recently diagnosed and started oral drug therapy (they were drug-naïve at the time the diagnosis was made) while 165 had been receiving anti-PD drugs. Our study was approved by the ethics committee of Tianjin Huanhu Hospital (2024-066). Written informed consent was obtained from all of the PD patients. Patients were recruited from September 2019 to December 2022 with the following criteria: (1) a clinical diagnosis of PD by an experienced neurologist major in movement disorders in line with MDS diagnostic criteria (Postuma et al., 2015); (2) age >18 years; (3) without severe cognitive impairment (Mini-Mental State Examination (MMSE) score > 24). The exclusion criteria were: (1) atypical PD symptoms or secondary parkinsonism; (2) history of cerebral infarction or hemorrhage, brain surgery, post-encephalitis, brain tumor, seizure, history of severe head trauma, or normal pressure hydrocephalus; (3) treatment with antipsychotic, immunosuppressant, or other drugs that may affect evaluation. A total of 635 patients were enrolled in the movement disorder clinic of our hospital and registered in the National Neurodegenerative Big Data Platform. For some patients, motor symptoms deteriorated rapidly, levodopa was not effective in treatment, or combined with fast-progressing dementia, eye movement disorder, ataxia and other symptoms, for these patients, we analyzed the results of magnetic resonance image, electrophysiological examination, cerebrospinal fluid discharge test, genetic testing and other examination results, and at the same time analyze the patient's previous data to modify the diagnosis. Among them, 23 patients were diagnosed with multiple system atrophy (MSA), 13 with Progressive supranuclear palsy (PSP), 5 with Dementia with Lewy bodies (DLB), 3 with Spinocerebellar ataxia (SCA), 4 with Essential tremor (ET), and 2 with Normal pressure hydrocephalus (NPH). And the other patients were excluded due to unwillingness to finish all the evaluations, loss of follow-up, missing data, etc. So, 320 patients were enrolled to our study.

### Procedures

Patient characteristic information was collected from all study participants. The clinical characteristics relevant to the analysis included time from symptom onset to drug treatment and Unified Parkinson's Disease Rating Scale (MDS-UPDRS). MDS-UPDRS-III was evaluated as the total score and subscores for tremor (sum of items 15 to 18), bradykinesia (sum of items 4 to 8 and 14), rigidity (item 3), and postural and gait impairment (sum of items 9 to 13) (Murakami et al., 2021). Patients were divided into treated (*N* = 165) and drug-naïve (*N* = 155) groups. The groups were sub-divided according to the ratio of tremor score to postural instability and gait disorder (PIGD) score derived from the MDS-UPDRS score, and categorized as either tremor dominant (TD) or non-tremor dominant (non-TD) subtypes (Jeong et al., 2021). The ratio of TD patients was ≧1.15, while the ratio of non-TD patients was <1.15. The MMSE and Montreal Cognitive Assessment (MOCA) were used to assess global cognitive abilities, and the Hamilton Anxiety Scale (HAM-A) and Hamilton Depression Scale (HAM-D) were used to assess psychological status. The 39-item Parkinson's Disease Questionnaire (PDQ-39) (Chen et al., 2017) was applied to assess the activity of daily living while Rapid Eye Movement (REM) Sleep Behavior Disorder Questionnaire-Hong Kong (RBDQHK) was utilized to assess REM sleep behavioral disorder (RBD) (Wang et al., 2015). All of the tests were performed in OFF state. The state of PD patients 72 h after discontinuing oral dopamine receptor agonists and 12 h after discontinuing oral levodopa preparations was considered as “OFF state” (de Souza Fortaleza et al., 2017; Dong et al., 2023). Levodopa equivalent daily dose (LEDD) was calculated by multiplying the daily Levodopa dose by the conversion formula widely used in previous studies (Zhang et al., 2021). LEDD (mg/d) calculation method = Levodopa standard tablet ^*^1+ levodopa controlled release tablet^*^0.75+ Entacapone ^*^0.33+ pramexole ^*^100+ Selegiline hydrochloride ^*^10+ resagiline ^*^100+ Piribedil^*^1+ amantadine ^*^1+ Rotigotine ^*^30+ Ropiniro^*^20 (Tomlinson et al., 2010).

### Statistics

Statistical analyses were performed using SAS9.4. The *T*-test was used for baseline demographic comparison between groups, and the significance level was 0.05. The Spearman's rank correlation coefficient was calculated and the number of clinical features being compared was adjusted. To determine the effects of treatment initiation time on motor and non-motor symptoms scores in PD, univariate regression analyses and multivariate regression analyses were performed to adjust age, sex, disease duration, age at symptoms. The corrected significance level was 0.05.

## Results

Clinical characters of enrolled patients are summarized in Table 1. The mean (standard deviation) age was 66.0 (8.7) years old in treated group vs. 65.1 (8.2) years old in *de novo* group (*p* = 0.31). The age at symptom onset in treated group was 59.2 (8.8) years old while the age at symptom onset was 62.9 (8.1) years old in *de novo* group (*p* < 0.01). In treated group the length of disease duration was 81.9 (44.5) months vs. 25.0 (20.8) months in *de novo* group (*p* < 0.001). Education level of the two groups were not significantly different (0.53). The mean time between PD symptoms onset and the initiation of drug treatment was 31.0 (22.5) months while the mean time between PD symptoms onset and the initiation of drug treatment in *de novo* group was 20.4 (18.6) months (*p* < 0.01). The LEDD in treated group was 592.7 mg/d. In treated group, 93 patients were TD and 72 were non-TD. In *de novo* PD group, 81 patients were TD and 74 were non-TD. The proportion of TD was higher in treated group, but the difference did not reach statistical significance (*p* = 0.46). Patients in the treated group had a statistical significantly longer disease duration and a higher MDS-UPDRS III score (*p* < 0.01). There were no significant differences in sex between the two groups (*p* = 0.58). There were no statistical differences betwee the proportion of hypertension (0.28), diabetes (0.40), Cardiovescular and cerebalvascular disease (0.26) or smoke status (0.67) (Table 1). The vast majority of patients in all treatment groups received either levodopa (here included dopa serazid sustained release tablets, carlevodidopa controlled release tablets, the same below) or levodopa combined with COMT-I. The frequency of dopa preparation is Tid or QID. 116/165 patients received DA, including pramipexole, ropinirol, rotigotine, and pibedil; The frequency of DA (including pramipexole and ropininile) in ordinary dosage forms was Tid, and the frequency of DA (including pramipexole extended-release tablets, Ropininile extended-release tablets, and Rotigotine patch) in sustained-release forms was usually Qd. Monoamine oxidase inhibitors (including selegiline and resagiline) were usually Qd in 23/165 patients.

**Table 1:** Demographic and clinical profiles of PD patients.

**Clinical characters**	**Treated group (N = 165)**	***de novo* PD group (*N =* 155)**	***p***
Age, mean (SD) [range]	66.0 (8.7) [39-83]	65.1 (8.2) [40-84]	0.31
Age at onset, mean (SD)	59.2 (8.8)	62.9 (8.1)	<0.01
Male (%)	85 (51.5%)	75 (48.4%)	0.58
Disease duration (months), mean (SD)	81.9 (44.5)	25.0 (20.8)	<0.001
TD (%)	93 (56.4%)	81 (52.3%)	0.46
Non-TD (%)	72 (43.6%)	74 (47.7%)	
Education level, mean (SD)	9.8 (3.6)	9.6 (3.7)	0.53
Time between symptom onset and treatment initiation (months), mean (SD)	31.0 (22.5)	20.4 (18.6)	<0.01
MDS-UPDRS III, mean (SD)	45.5 (16.2)	28.8 (15.3)	<0.01
LEDD, mean (SD)	592.7 (294.1)	NA	NA
Treated time (SD)	NA	50.9 (33.0)	NA
Hypertension (%)	94 (56)	79 (51)	0.28
Diabetes (%)	33 (20)	24 (16)	0.30
Cardiovescular and cerebalvascular disease (%)	56 (34)	55 (32)	0.76
Smoke (%)	59 (36)	50 (33)	0.85

MDS-UPDRS, Unified Parkinson's disease rating scale; NMSS, non-motor symptoms scale; LEDD, Levodopa equivalent daily dose; TD, tremor dominated; Non-TD, non-tremor dominated; SD, standard deviation.

**Figure 1:**
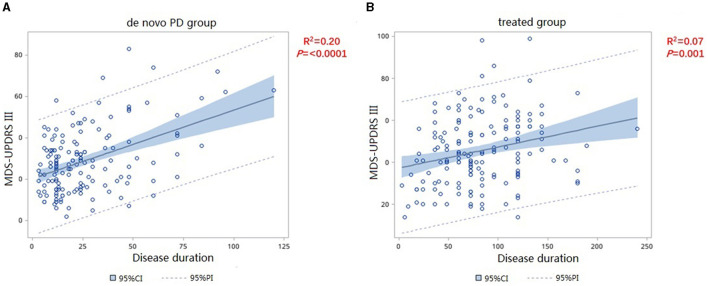
Correlation of MDS-UPDRS-III change and disease duration in *de novo* **(A)** and treated **(B)** patients.

**Figure 2:**
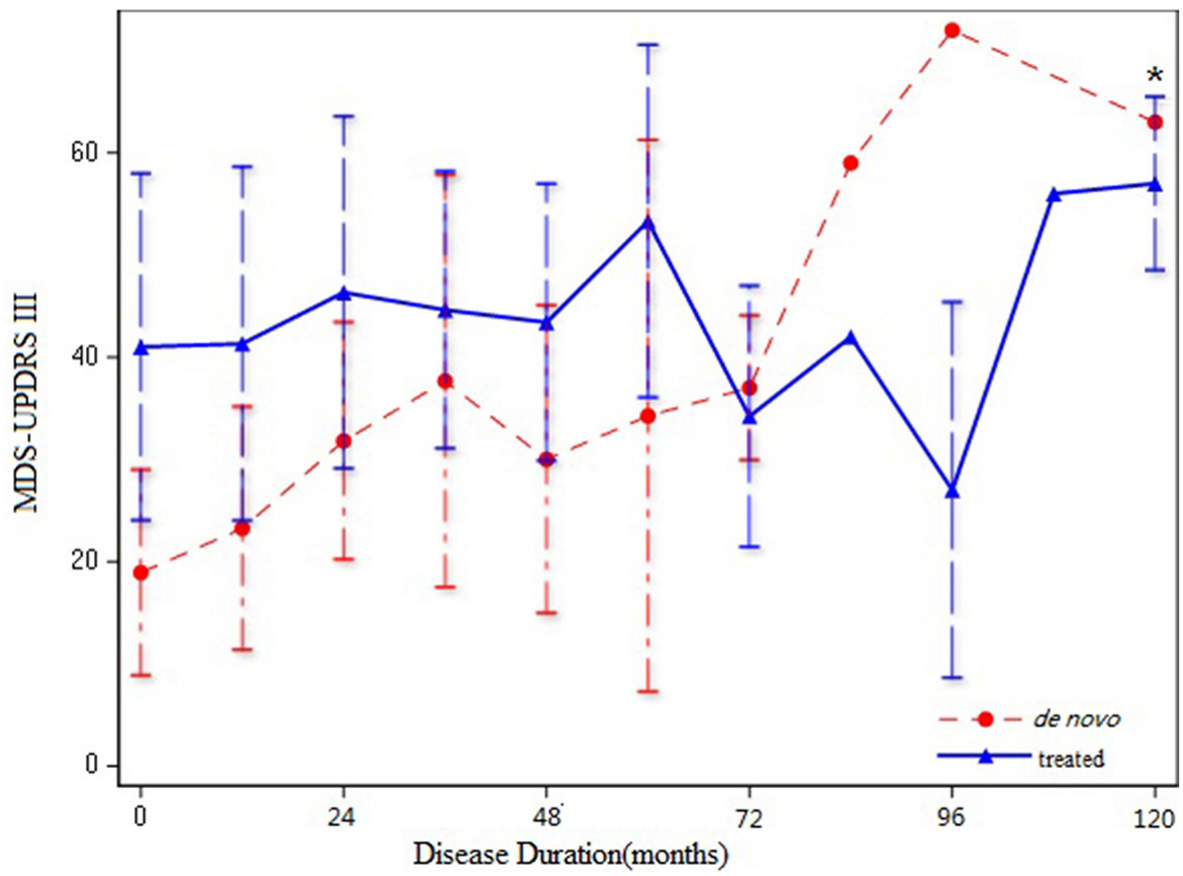
Line chart of MDS-UPDRS III score in treated group and drug-naïve group *p < 0.01.

We included the MDS-UPDRS-III score as the dependent variable to assess the severity of motor symptoms. Since PD progresses gradually over time, we included disease duration as an independent variable and analyzed the correlations with in MDS-UPDRS-III score between the two groups. The slope of *de novo* PD group is significantly higher than that of the treated group (F = 13.21, *p* = 0.0003) (Figure 1), which shows the variation tendency of score was more obvious in the *de novo* group than in the treated group. After adjusting for age, sex, age at onset, and disease duration, the slope of the two group were still statistically different, indicating that the MDS-UPDRS-III score increased faster in patients in the *de novo* group with the same disease duration as patients in the treated group (F = 8.7, *p* = 0.0034) (Table 2). We merged the curves of the two groups on one graph to show the difference in UPDRS III scores between the two groups over the same duration of disease. The UPDRS III scores in de novo group were significantly higher than in treated group (*p* < 0.01) (Figure 2).

**Table 2:** Difference of the slope between the two groups after adjusted age, sex age at onset and disease duration.

**Source**	**DOF**	**Mean square**	**F**	**Pr > F**
Numerator	1	1872.99348	8.7	0.0034
Denominator	312	215.16371		

DOF, Degree of Freedom.

**Figure 3:**
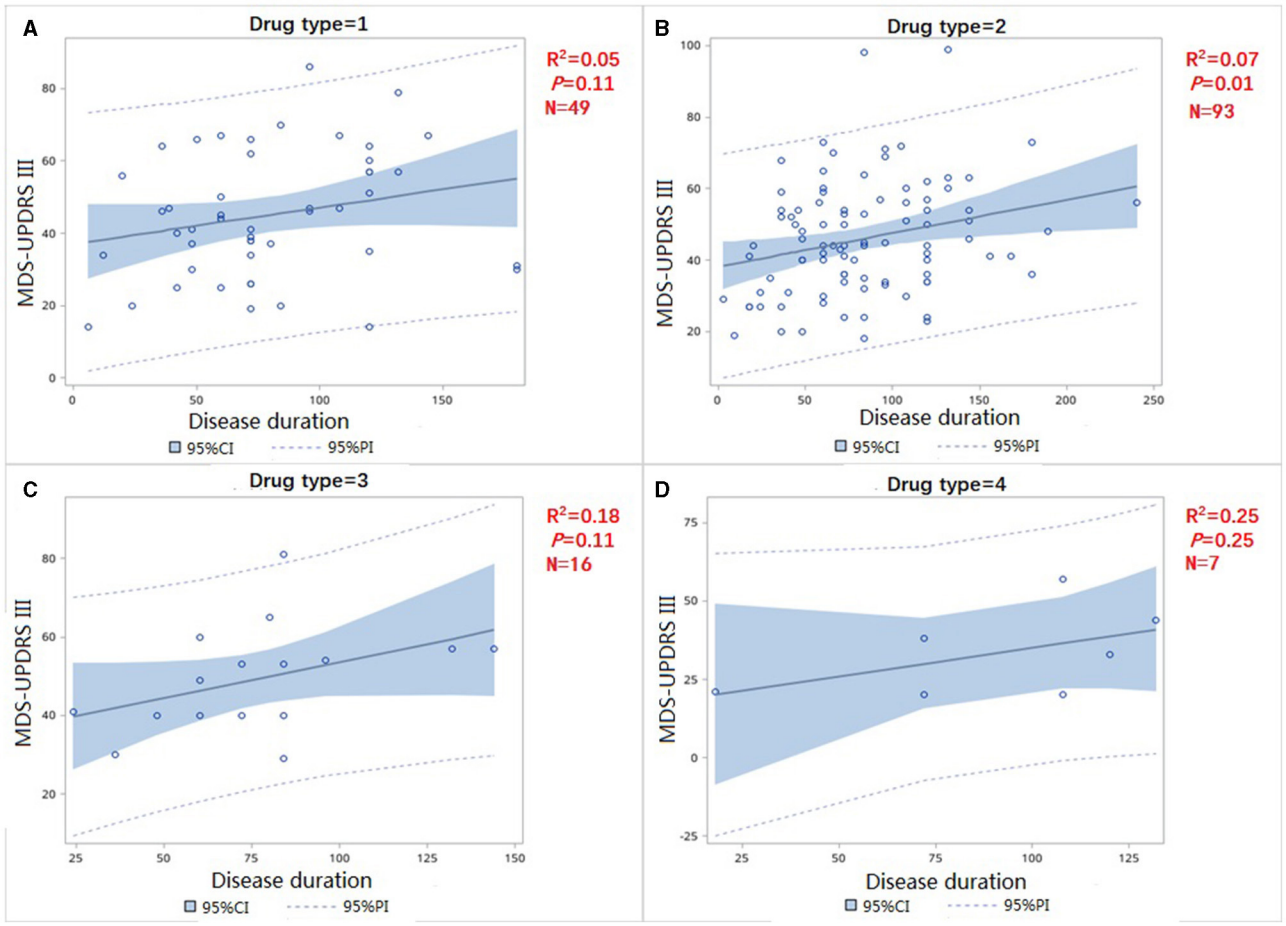
Correlation of MDS-UPDRS-III change and disease duration in treatment group **(A)** use levodopa preparations or levodopa preparations combined with COMT-I **(B)** use levodopa preparations combined with dopamine receptor agonists **(C)** use levodopa preparations combined with dopamine receptor agonists **(D)** use dopamine agonist only.

We divided patients in the treated group into four subgroups, with the first group (drug type = 1, Figure 3A) representing the administration of levodopa preparations or levodopa preparations combined with COMT-I. The second group (drug type = 2, Figure 3B) indicated the use of levodopa preparations combined with dopamine receptor agonists; The third group (drug type = 3, Figure 3C) indicated the use of levodopa preparations with monoamine oxidase inhibitor with or without DA; The fourth group (drug type = 4, Figure 3D) represented dopamine agonist alone (all patients in this group received pramipexole). The slope of the four group were not statistically different (F = 0.06, *p* = 0.8148) (Figure 3), indicating that the type of therapeutic drugs may not be used as an independent variable in the analysis.

**Figure 4:**
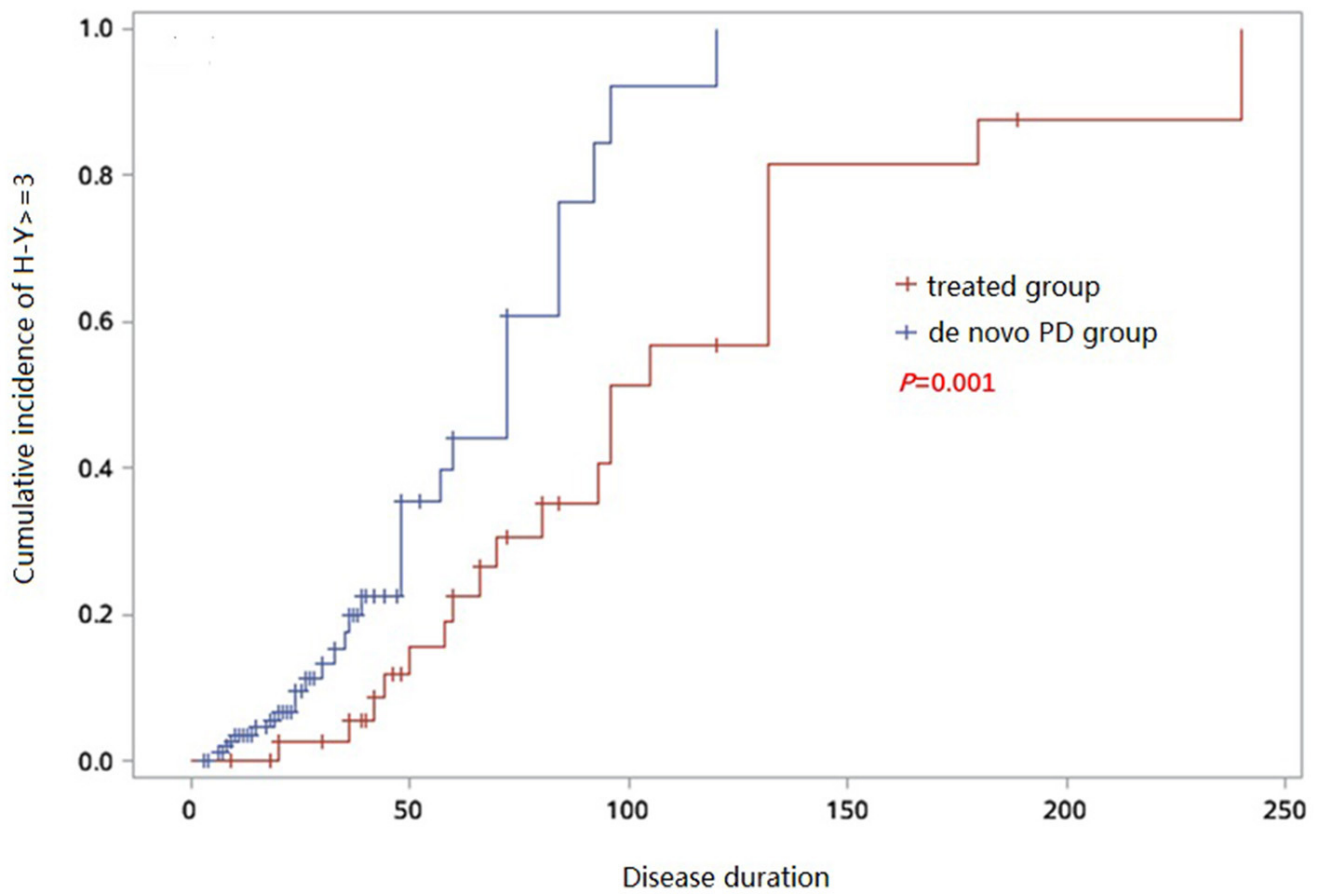
Kaplan–Meier analysis of cumulative incidence to H-Y ≥ 3 of *de novo* and treated PD groups.

PD patients with a Hoehn and Yahr (H-Y) scale score ≥3 are generally considered to be in the advanced stage (16). We generated a Kaplan-Meier survival curve to analyze the cumulative incidence of progression to H-Y score 3 in the two groups and concluded that the cumulative incidence of patients. By fitting the curve, the cumulative incidence of progression to H-Y score 3 in *de novo* PD group over disease duration was 39.7% in 50months and 92.2% in 100 months, while in treated group such cumulative incidence was 15.5% in 50 months, 51.4% in 100 months and 81.5% in 150 months. The cumulative incidence of progression to H-Y score 3 was higher in *de novo* group than that in the treated group (*p* = 0.01) (Figure 4), suggesting that untreated patients were more likely to progress to the advanced stages.

The results of the regression analysis performed to determine the effect of the timing of the initiation of drug treatment on MDS-UPDRS scores are shown in Table 3. The dependent variable included in the regression model was the MDS-UPDRS-III score and the independent variables included onset symptom (TD or non-TD), time between symptom onset and treatment initiation (months), age, sex, and disease duration (months). The results show that the total regression model was statistically significant (F = 56.53, *p* < 0.01). Results in Table 3 show that the adjusted R^2^ was 0.2895, indicating that these five variables together explained 28.95% of the total variation in the MDS-UPDRS-III score for motor symptoms. The results in Table 4 show that after other variables were screened out, the adjusted R^2^ was 0.2840, indicating that the disease duration and onset symptom could explain 28.4% of the total variation in the MDS-UPDRS-III score of motor symptoms. The obtained multiple linear regression equation was:


$$\begin{array}{l} \hat{\text{Y}}=0.28651\times \text{ Disease duration}+21.66428\times \text{ symptom} \end{array}$$

**Table 3:** Multivariate regression analysis for time between symptom onset and treatment initiation with and without screening out variables.

**Variate**	**Screen out**	**DOF**	**Standard error**	**t**	***p***	**Standardized estimates**	**VIF**
Intercept	Yes	1	7.73543	−1.44	0.1502	NA	NA
Time between symptom onset and treatment initiation	Yes	1	0.05633	1.30	0.1933	NA	NA
Age	Yes	1	0.10712	1.82	0.0698	NA	NA
Sex	Yes	1	1.80199	0.96	0.3404	NA	NA
Symptoms onset	Yes	1	1.83859	0.06	0.9526	NA	NA
Disease duration	Yes	1	0.07165	3.00	0.0030	NA	NA
Outcomes of model fitting		R^2^= 0.2895					
Intercept	No	1	3.67438	−0.16	0.8759	0	0
Disease duration	No	1	0.05440	5.27	<0.0001	0.30459	1.30812
Symptom	No	1	2.03756	10.63	<0.0001	0.61494	1.30812
Outcomes of Model Fitting		R^2^ = 0.2840					

DOF, Degree of Freedom; VIF, Variance Inflation Factor.

**Table 4:** Univariate regression analysis of time between symptom onset and treatment initiation for *de novo* patients.

**Variate**	**Parameter estimation**	**Standard error**	**t**	***p***	**R^2^**
Symptom onset	−1.92078	2.51290	−0.76	0.4459	0.00401
Time between symptom onset and treatment initiation	0.38043	0.05911	6.44	<0.001	0.21306
Age	0.16110	0.14465	1.11	0.2671	0.00804
Sex	0.21628	2.46512	0.09	0.9302	0.00005031
Disease duration	0.28007	0.05164	5.42	<0.001	0.16123

We analyzed the data from the 155 *de novo* patients enrolled in this study separately. Univariate regression analysis for time between symptom onset and treatment initiation on MDS-UPDRS-III scores are shown in Table 5. The dependent variable included in the regression model was MDS-UPDRS-III score and the independent variables included onset symptom (TD or non-TD), time between symptom onset and treatment initiation (months), age, gender, and disease duration (months). The results show that the total regression model was statistically significant (F = 37.76, *P* < 0.01). The adjusted R^2^ of 0.2012 indicates that the time between symptom onset and treatment initiation explained 20.1% of the total variation in the MDS-UPDRS-III score for motor symptoms. The results in Table 5 show that after other variables have been screened out, both intercept and treatment initiation time had statistical significance. The multiple linear regression equation obtained is as follows:


$$\begin{array}{l} \hat{\text{Y}}=20.8831+0.36633\times \text{time between symptom onset and }\\ \text{ treatment initiation}.\text{ The results indicate that patients with}\\ \text{ delayed treatment initiation had higher}\\ \text{ MDS-UPDRS-III scores}. \end{array}$$

**Table 5:** Multivariate regression analysis of time between symptom onset and treatment initiation by screening out variables for *de novo* patients.

**Variate**	**Parameter Estimation**	**Standard error**	**t**	***p***	**Standardized estimates**
Intercept	20.88310	1.67678	12.45	<0.001	0
Time between symptom onset and treatment initiation	0.36633	0.05961	6.15	<0.001	0.45456
Outcomes of model fitting		R^2^ = 0.2012			

**Figure 5:**
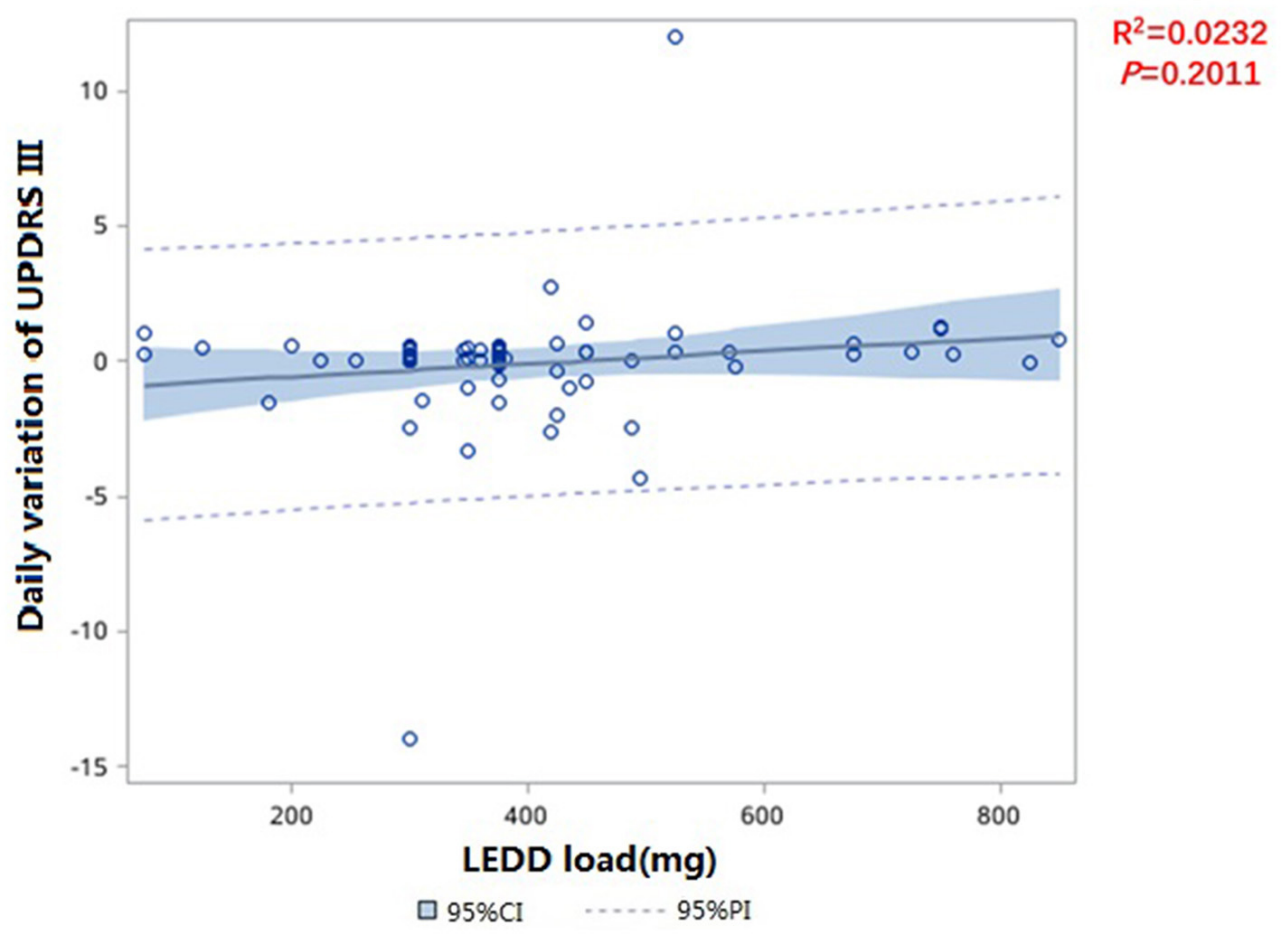
Fitting curve of daily variation in UPDRS III and LEDD load.

These 165 *de novo* PD patients were drug-naive and started dopaminergic replacement therapies after evaluations. We evaluated UPDRS III scores after treatment. Since patients were followed up at different times, we used the daily variation in UPDRS III to eliminate any bias associated with different follow-up times. There was no significant statistical difference in the slope of UPDRS III after treatment (*p* = 0.20), which showed that treatment did not slow the disease progression (Figure 5).

## Discussion

The main finding of this study is that the treatment group had a lower MDS-UPDRS-III score than drug-naïve group as the disease duration increased, which is consistent with the findings of previous studies (Verschuur et al., 2019). This was done in the Levodopa OFF state (72 h after stopping treatment) to evaluate the disease without replacement therapy. We found that the time of treatment initiation of PD patients was particularly important, and could explain explained 20.1% of the total variation in the MDS-UPDRS-III score for motor symptoms in drug-naïve patients. This suggests that the precise treatment timing for PD is important and early dopaminergic treatment should be recommended. We also concluded that the cumulative incidence of patients progressing to advanced stages was higher in the *de novo* PD group, so it is particularly important to identify early symptoms of PD to improve correct diagnosis and early treatment.

The LEAP study reported a similar incidence and severity of motor complications in PD patients both in early-initiated and delay-initiated groups (Verschuur et al., 2019). Previous studies demonstrated that in drug-naïve PD patients, delaying treatment for 6 months could provide insights into motor symptoms progression (Schapira et al., 2010, 2013; Lohle et al., 2014), and motor scores of MDS-UPDRS have been reported to decline by 5.1 % annually (Schrag et al., 2007). The PROUD study suggested that delaying the start of the drug treatment by 6 months might did not affect disease symptoms in PD patients (Schapira et al., 2010). There is evidence that early dopaminergic therapy might partially restore basal ganglia function, which supports physiological compensatory mechanisms and can delay the loss of dopaminergic neurons which causes motor symptom progression in PD patients (Lang et al., 2020).

In the past few decades, dopamine replacement strategies have been the most effective therapy for PD patients. The side effects of Levodopa-induced motor fluctuations and dyskinesia, as well as the impulse control disorder (ICD) of DA are the main reasons for the hesitancy to give patients Levodopa in the early stages of PD (Olanow, 2015). The effect of disease severity and side effects have been highlighted by recent studies that evaluated patients in Ghana and Italy (Cilia et al., 2014). Pharmacotherapy was started earlier in Italy (mean course of disease, 2.4 years in Italy vs. 4.2 years in Ghana; *p* = 0.001). Disease duration at the occurrence of motor fluctuations and dyskinesias was similar in the two populations. In multivariate analysis, disease duration (*p* = 0.04) and levodopa daily dose (mg/kg of body weight) (*p* = 0.019) were associated with motor complications, while the disease duration at the initiation of levodopa (*p* = 0.60) was not. The researchers indicated that motor complications were not associated with the initiation of dopaminergic supplementation, but with longer courses of PD disease and higher treatment doses. In clinical work, some patients worried that early initiation of treatment may lead to the decline of drug efficacy later in the course of disease, so they were hesitate to initiate treatment. In fact, previous research has demonstrated that older age at onset, higher baseline H-Y stage, and severe cognitive impairment have a negative impact on the prognosis of PD (Baba et al., 2022; Wang et al., 2022). Therefore, we suggest that PD patients should adopt early dopamine replacement treatment for PD treatment, which can be beneficial for the effective treatment of PD disease.

For PD patients, treatment is symptomatic. Drugs that enhance intracerebral dopamine concentrations or stimulate dopamine receptors remain the mainstay of treatment for motor symptoms. To our knowledge, all dopaminergic therapy drugs can only alleviate PD symptoms, but cannot delay the progression of the disease, cure it, or reverse its neurodegenerative effects (Armstrong and Okun, 2020; Liu et al., 2023). In our study, the slope of UPDRS III after treatment was not significantly difference from baseline, indicated that motor symptoms may be improved by treatment, but did not slow the disease progression, which was in accordance with former studies (Johnson et al., 2013; Barbagallo and Quattrone, 2019). Since the time of follow-up was relatively short, continued long-term follow-up is warranted to evaluate the rate of disease progression.

In our study, we found that symptom onset was not significantly affected by the total variation in the MDS-UPDRS-III score in the subgroup analysis of this study, which conflicts with the results of previous studies (Kohat et al., 2021; Skidmore et al., 2022). One possible explanation is that most of the enrolled patients were H-Y stage 1 and 2, and the disease duration was relatively short. Therefore, future studies with a larger sample size should be carried out to further investigate the relationship between initiation of dopaminergic replacement treatment and PD disease progression.

Since the use of technology, especially artificial intelligence has been increasing over the past decade (Mukhopadhyay et al., 2019). Technological advances can improve the accuracy of diagnosis and treatment and provide neurologists with effective and efficient tools in a timely manner during the long-term management of PD. As the acquisition of large clinical data is growing, by using the results from studies like our study, neurologists and machines can work cooperatively to establish quantitative models to predict the health outcome and prognosticate disease procedure, which may better promote the research on the disease mechanism of movement disorders (Mofatteh, 2021).

We believe that the strength of this study, in contrast to previous studies (Cilia et al., 2014), to be the inclusion of PD patients who were identified by clinical diagnosis, and are therefore representative of population rather than having disease caused by specific genetic or environmental factors. Our finding is that, in this population, pharmacotherapy should be initiated early (i.e., soon after diagnosis) to help PD patients maintain their ability to have high quality of life and better long-term health to alleviate disease burden (Martinez-Martin et al., 2019).

It is important to note that this study has several limitations. First, the sample size of our PD cohort is relatively small. Second, the treatment group consisted of patients who were already undergoing therapy for PD for varying lengths of time, which introduces a degree of heterogeneity into the study population. Long-duration response to levodopa (LDR) derives from prolonged administration of L-dopa and persists for hours to days after treatment discontinuation independently of the peripheral pharmacokinetics (Zhang et al., 2021). So The effects of the drug may not be completely eliminated by 72 h withdrew of treatment. Ultimately, the decision of when to initiate dopamine replacement strategies needs to be made by the patient in consultation with their physician. It is also necessary to discuss the potential benefits and side effects of medication in detail with PD patients. More research is needed to determine what factors should be considered in the decision for when to start intervention.

In conclusion, our study reports that the severity of PD disease in terms of MDS-UPDRS motor section score is correlated with the time of treatment initiation, which indicated that there is no reason to delay the initiation of adequate dopaminergic replacement therapy in patients with Parkinson's disease. Thus, recognizing early-stage symptoms of PD and early diagnosis are vital to providing effective treatment.

## Data availability statement

The original contributions presented in the study are included in the article/supplementary material, further inquiries can be directed to the corresponding author.

## Ethics statement

The studies involving humans were approved by Ethics Committee of Tianjin Huanhu Hospital. The studies were conducted in accordance with the local legislation and institutional requirements. The participants provided their written informed consent to participate in this study.

## Author contributions

XL: Formal analysis, Methodology, Writing – original draft. Z-yD: Writing – review & editing. MD: Writing – review & editing, Data curation. LC: Formal analysis, Funding acquisition, Methodology, Project administration, Resources, Visualization, Writing – review & editing.
